# Effectiveness of mental simulations on the early mobilization of patients after cesarean section: a randomized controlled trial

**DOI:** 10.1038/s41598-021-02036-1

**Published:** 2021-11-22

**Authors:** Anna Prokopowicz, Katarzyna Byrka

**Affiliations:** 1 Division of Midwifery and Gynaecological Nursing, Department of Nursing and Obstetrics, Faculty of Health Sciences, Wroclaw Medical University, ul. Kazimierza Bartla 5, 50-996 Wrocław, Poland; 2 Department of Gynecology and Obstetrics, University Hospital in Wroclaw, Wrocław, Poland; 3 Faculty of Psychology in Wroclaw, SWPS University of Social Sciences and Humanities, Wrocław, Poland

**Keywords:** Psychology, Health care

## Abstract

We aimed to investigate whether psychological intervention (single mental simulation) among women after cesarean surgery (CC) can affect their willingness to verticalize, actual verticalization, and the duration of the first mobilization. In this prospective randomised, controlled study, 150 women after CC were divided into 3 groups: experimental group with process-simulation with elements of relaxation, experimental group with outcome-simulation with elements of relaxation and control group with elements of relaxation only. After a 5-h stay in the post-operative room, women listened to a recording with a stimulation. Pain and anxiety of verticalization were measured before and after listening to the recording and after verticalization. Almost 12% more patients verticalized in the process-simulation group than in the control group. Percentages of mobilized patients were: 39.4% the process-simulation group; 32.8% in the outcome-simulation group; 27.7% controls (p = 0.073). Mobilization was 5 min longer in the process-simulation group then in control (p < 0.01). Anxiety after the simulation was a significant covariate of the willingness to verticalize, actual verticalization and time spent in mobilization. We conclude that a single mental simulation can effectively motivate patients for their first verticalization after CC. Perceived anxiety before verticalization may affect the effectiveness of interventions, so we recommend to check it at the postoperative care.

ClinicalTrials.gov Identifier: NCT04829266.

## Introduction

Multiple studies have shown, that early mobilization positively affects the recovery process after surgery^1–5^. Effective intervention techniques that would enhance women’s motivation for early mobilization on day 0 after cesarean surgery, despite the perceived anxiety of verticalization, are on demand for at least two reasons. First, a woman is the patient that requires care after an operation. Second, she is a mother who wants to be able to take care of her newborn baby several hours after the surgery. In this study, we explored mental simulations as techniques that may enhance patients' motivation for the first verticalization after cesarean section. The effectiveness of such theory-based psychological interventions on day 0 after cesarean surgery has not been studied before.

According to the Enhanced Recovery After Surgery (ERAS) protocol, commonly applied in practice multimodal analgesic therapy in the perioperative period allows for a maximum reduction of pain during and after surgery. Nonetheless, low levels of pain do not guarantee the elimination of the anxiety related to pain anticipated by patients. A previous study on anxiety and pain in women on day 0 after their cesarean section revealed that patients who experienced almost no pain because of analgesic treatment after surgery still declared anxiety of verticalization as measured on the numerical rating scale (NRS) ^6^. It is known at the same time, that anxiety negatively affects people’s cognitive and motivational functioning by inducing avoidance behaviors^7^.

Non-pharmacological interventions can improve patient’s physical and mental conditions after surgery and subsequently accelerate the patient’s rehabilitation. So far, studies have focused on different non-pharmacological methods of reducing pain and anxiety after cesarean surgery, such as acupressure, aromatherapy, body massage, talking and conversing or music and relaxation^8–16^. An often-mentioned effective technique applied after surgery is relaxation. It reduces pain and anxiety and is often recommended in protocols for early postoperative care^15^. For example, a longitudinal study with a Benson relaxation technique decreased postoperative pain^12^. Bitzer proposed that in obstetrics in stressful situations, the use of relaxation techniques focusing on the reformulation of catastrophic thinking into task-oriented thinking is beneficial for a patient^16^.

With this study, we focus on mental simulations, one more non-pharmacological technique that has not been tested as means to motivate patients for early mobilization, but has been found effective in other contexts. Mental simulations are intentionally recalled images from the past or representations of future hypothetical events^17^. In contrast to semantic and verbal representations with a higher level of abstraction, mental simulations involve detailed representations of a specific or hypothetical reality^18^. A mental simulation of motor actions may be functionally equivalent to motor training before performing the activity. The motor simulation also translates into time and amount of effort used on the activity at the motor level^19^. Mental simulations can play a significant role in self-regulation and planning^17,20,21^. Training with mental simulations can improve endurance in sports^22^ and the efficiency of damaged muscles^23^.

Not all mental representations are equally effective. Visualization of the processes of committed, goal-oriented action is more effective in achieving the goal than focusing on the positive outcomes of this action^17^. Taylor et al. divided mental simulations into three groups: (1) process simulation—imagination of the structure of a planned activity, (2) outcome simulation—imagination of the desired goal, and (3) rumination—negative thinking, often generating suffering and pain, blocking, and hindering action. The mental process simulations have a regulatory function. They prepare individuals who aim to achieve a goal, for a tangible action in safe conditions, in their mind^24^. According to the classification of coping styles developed by Endler and Parker, the process simulation can be equated to the most effective, adaptive style, which is focused on the future task^24,25^. The effectiveness of the process simulation, built on the conceptualization of overcoming potential disturbances while achieving the goal, has been demonstrated in various research areas (e.g., education, slimming, consumer behavior)^26–28^. The outcome simulations have been linked to an emotion-centered style with a tendency to wishful thinking^24,25^. So, they may be less effective in situations affectively loaded, such as a child’s birth. The use of simulation elements of the process of the first verticalization after surgery combined with elements of a free Benson’s relaxation^29^ may have practical application in the postoperative care of patients after cesarean section.

### Research goals

This study investigated whether the use of psychological intervention in the form of a single mental simulation among women after cesarean surgery would affect their willingness to verticalize, actual verticalization, and the duration of the first mobilization.

Specifically, we hypothesized that the willingness to verticalize would be the greatest in the process-simulation group than in the outcome-simulation group and then in the control group. In the process-simulation group, we also expect the highest number of patients to verticalize. Relatively fewer patients should verticalize in the outcome-simulation group and the least in the control group. Finally, the duration of the first mobilization will be the longest in the process-simulation group than in the outcome-simulation group and then in the control group. We predicted that anxiety and experienced pain will also play the role and will affect the effectiveness of psychological interventions. Therefore, we additionally controlled for these variables.

## Methods

### Participants and settings

Between December 15, 2017, and May 27, 2018, 150 women were examined on day 0 after cesarean section. Inclusion criteria were the following: signed informed consent for participation in the study; age > 18 years; conduction of anesthesia during Cesarean section; undergoing the same analgesic therapy in the postoperative period; no orthopedic, neurological, and psychiatric dysfunctions; being qualified to mobilization by the medical staff; and fluent at Polish in speaking and writing. Notably, 150 patients fulfilled the mentioned criteria. The mean age with standard deviation of participants was 32 ± 4.6 year. The youngest studied person was 20 years old, the oldest 48 years old. For more details concerning the sample see Appendix.

As this study was the first one testing mental simulations in perioperative care, we had no grounds for specific effect sizes, we thus assumed medium effect sizes (Cohen’s w = 0.30), power (1-*β*) = 0.90, and used probability level *α* = 0.05. For these assumptions, we determined a sample size of at least *N* = 141 for the least powerful χ^2^.

### Interventions (design and course of the experiment)

All women were divided into three independent groups: (1) a control group with no process and outcome simulations, but with elements of relaxation; (2) an experimental group with process simulation with elements of relaxation; and (3) an experimental group with outcome simulation with elements of relaxation. An assignment to the study groups was based on the randomization pattern using the Research Randomizer Version 4.0 program^30^. MP3 recordings were assigned to patients in the order of arrival at the postoperative room. The women listened to the text using headphones about 10–15 min before verticalization (5 h after arrival in the postoperative room, about 6 h after anesthesia). In each group, the mental training lasted for about 10 min. The experimenter conducting the research was unaware before the end of the entire research process which text was recorded on which MP3. However, the given MP3 number was written on all questionnaires.

The study was conducted in the Postoperative Room of the Gynecology and Obstetrics Department at the University Clinical Hospital in Wroclaw, Poland. Directly after arriving at the postoperative room, the women became acquainted with the examination procedure and signed written informed consent for participation in the study. No fee was paid to participants. All questionnaires were completed anonymously. The study was approved by the Ethics Committee of the SWPS University of Social Sciences and Humanities in Wrocław, Poland (05P/12–2017). All methods were performed in accordance with the relevant guidelines and regulations applicable. The study was registered at the ClinicalTrials.gov (Identifier: NCT04829266; date of registration 02/04/2021).

Directly after arriving at the postoperative room, the women filled out the form of informed consent. Next, the level of anxiety as a permanent disposition was measured in the patients (T0 measurement). Five hours after arrival in the postoperative room (6 h after anesthesia and about 10–15 min before verticalization), the studied women listened to recordings using headphones, considering the previous assignment to the independent study groups: with process-visualization, with outcome-visualization, and with the fragment of the book. Patients marked the pain and anxiety of verticalization that they felt immediately before (T1 measurement) and after listening to the recording (T2 measurement). After listening to the recording, they also marked one of the options of the subjective willingness to verticalize on the questionnaire developed by the authors.

Each patient was verticalized or not according to her preference for verticalization at each stage of the study. After returning to bed, the patient reported the average pain she experienced during upright standing (T3 measurement). The subsequent measurements of the study variables are shown in Table 1.

**Table 1:** Sample size, means, standard deviations and confidence intervals in each group.

	Number of participants in the group	Mean	Standard deviation	Confidence intervals
Control group	43	4.16	1.21	3.81–4.52
Process simulation	55	4.55	1.05	4.24–4.86
Outcome simulation	52	4.27	1.24	3.95–4.59

### Questionnaires and tools (MP3)

The study used recordings developed by authors on an MP3 carrier played to patients via external headphones. All three recordings contained similar calming and relaxing commands (relating to calm breathing, relaxing, closing the eyes, activating the sensory senses in the imagination) and various mental simulation commands depending on the type of recording. All recordings were in Polish and had the same number of words and the same duration.

The recording of the control group (number 1) was neutral without the process visualization and the outcome activation. It contained a fragment of the book “Little Prince” by Antoine de Saint-Exupéry, including an order to imagine the elements of the world as seen through the eyes of the main character^31^. The recording of the process simulation (number 2) included the order to imagine subsequent elements of the process of mobilization. The recording of the outcome simulation (number 3) included an optimistic vision of returning to health and fitness. For the exact content of manipulations see Appendix.

The following observed variables were operationalized using the self-report method: pain, anxiety, and willingness to get up. Anxiety and pain were measured on the NRS from 0 to 10. Previous studies showed that the accuracy and reliability of these scales allow their use in scientific research^32,33^. On the NRS of pain, 0 denotes no pain, and 10 corresponds to the most severe pain imaginable. For the purpose of this study, when considering anxiety on the NRS, 0 denotes a complete absence of anxiety of verticalization, and 10 corresponds to the strongest anxiety of verticalization imaginable.

The studied women assessed their willingness to verticalize on a six-point scale (from 1 to 6). On this scale, they marked one of the points that corresponded to their status: 1—I am definitely not ready to get up and remove the catheter; 2—I do not think I am ready to get up and remove the catheter; 3—I think I am ready to get up but not to remove the catheter; 4—I think I am ready to get up, but the catheter can be removed after successful verticalization; 5—I think I am ready to get up and remove the catheter; 6—I am definitely ready to get up and remove the catheter. The development of such short scales that fit the specific clinical situation of the patient has already been used in the scientific literature^34,35^. The examiner noted the success or failure of mobilization of the patient in the patient’s records. The examiner noted the success or failure of the patient’s mobilization along with its duration (the start of the measurement was set at getting out of bed from a sitting position, and the end of the measurement was set at the patient’s return to bed). If the patient got out of bed and started walking (she took at least one step), she received a score of 1(success) in the questionnaire. If she got up, but did not take a single step returning to the sitting position, she received a score of 0. The duration of mobilization was measured in minutes.

### Ethics approval

The study was approved by the Ethics Committee of the SWPS University of Social Sciences and Humanities in Wrocław, Poland (05P/12-2017).

### Consent to participate

All women signed written informed consent for participation in the study directly after arriving at the postoperative room. No fee was paid to participants.

## Results

A univariate analysis of variance (ANOVA), the omnibus Kruskal–Wallis H test along with the Dunn’s post-hoc analyses with Bonferroni correction were conducted, logistic and ordinal regression models were developed. Statistical analysis was carried out with Statistical Package for Social Sciences IBM (SPSS-IBM), version 22 (SPSS Inc., Chicago, Illinois, USA).

### Effect of simulations on willingness to verticalize

In the first step, we tested the effectiveness of the simulation manipulation on the willingness to verticalize. A univariate analysis of variance (ANOVA) showed no significant effect of the manipulation, *F*(2, 149) = 1.42, *p* = 0.239, *r*^*2*^_*effect*_ = 2%. This means that independent of which group the patients were assigned, they were ready for verticalization to the same extent. Descriptive statistics on the willingness for verticalization in each group can be found in Table 1.

In line with our research goals, we ran additional analyses in which we controlled for anxiety experienced after the manipulation and pain. A univariate analysis of covariance (ANCOVA) with anxiety after the simulation and pain as covariates showed an insignificant main effect of the manipulation, *F*(2, 149) = 2.24, *p* = 0.110, *r*^*2*^_*effect*_ = 3%. Anxiety was a significant covariate and explained 19% of variance in the willingness to mobilize, *F*(1, 149) = 23.52, *p* < 0.001 *r*^*2*^_*effect*_ = 14%. Pain was not a significant covariate in the model, *F*(1, 149) = 2.24, *p* < 0.01 *r*^*2*^_*effect*_ = 0%. Overall, the willingness to verticalize was mostly affected by anxiety measured after the manipulation and to a much lesser extent by pain.

### Effect of simulations on early verticalization

**Figure 1:**
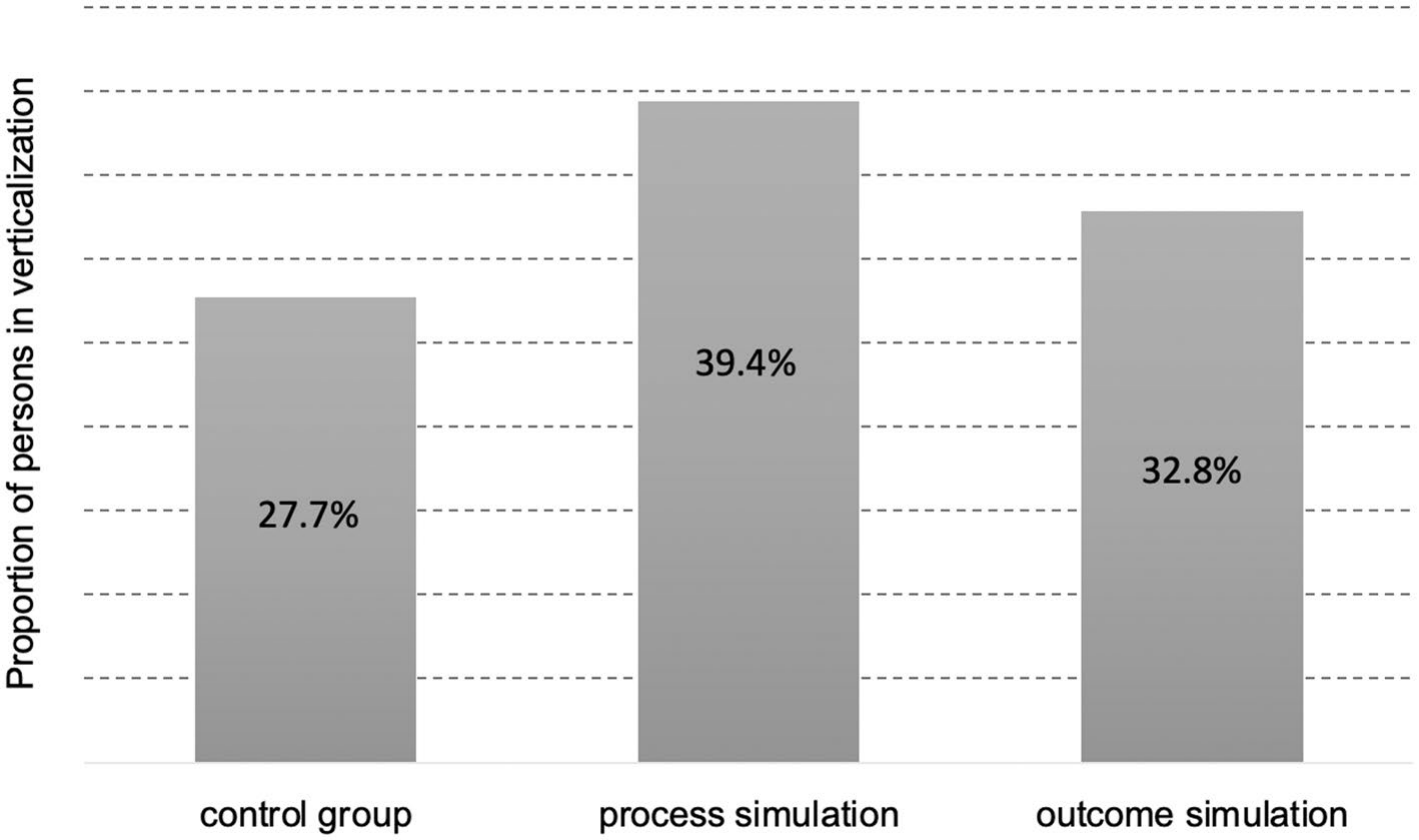
A proportion of verticalized patients in the control, process-simulation, and outcome-simulation groups.

Next, we tested the effect of manipulation on whether the patients mobilized or not six hours after the cesarean cut. We found a significant difference between the groups in the omnibus test using the likelihood ratio test, χ^2^(2, *N* = 150) = 6.43, *p* < 0.05. Pearson’s chi-square was marginally insignificant, χ^2^(2, *N* = 150) = 5.25, *p* = *0.073*, φ = 0.19. In line with our predictions, the largest proportion of mobilized patients was observed in the process-simulation group (39.4%, *n* = 54), then in the outcome-simulation group (32.8%, *n* = 45), and finally in the control group (27.7%, *n* = 38). When examined closer, patients in the process-simulation group verticalized significantly more likely than the expected value (adjusted residual = 2.3). A proportion of verticalized patients in each study group is shown in Fig. 1.

We ran an additional logistic regression model in which we tested the role of anxiety after the simulation and pain on verticalization. We entered dummy coded manipulation, anxiety after the simulation, and pain as predictors. Verticalization was an outcome variable. The manipulation variable was dummy coded into two variables, that is X_1_—process simulation (1) vs. outcome simulation and the control group (0) and X_2_—outcome simulation (1) vs. process simulation and the control group (0). The logistic regression revealed that process simulation was a marginally insignificant predictor (X_1_), *W* = *3.68, p* = *0.055*. Anxiety was a significant predictor, *W* = *6.58, p* < 0.01, but pain was not, *W* = *4.81, p* < *0.05.*

Overall, consistent with our hypotheses, process simulation was the most effective in motivating patients to verticalize. Actual verticalization was affected by anxiety, but not experienced pain.

### Effect of simulations on time spent during mobilization

**Figure 2:**
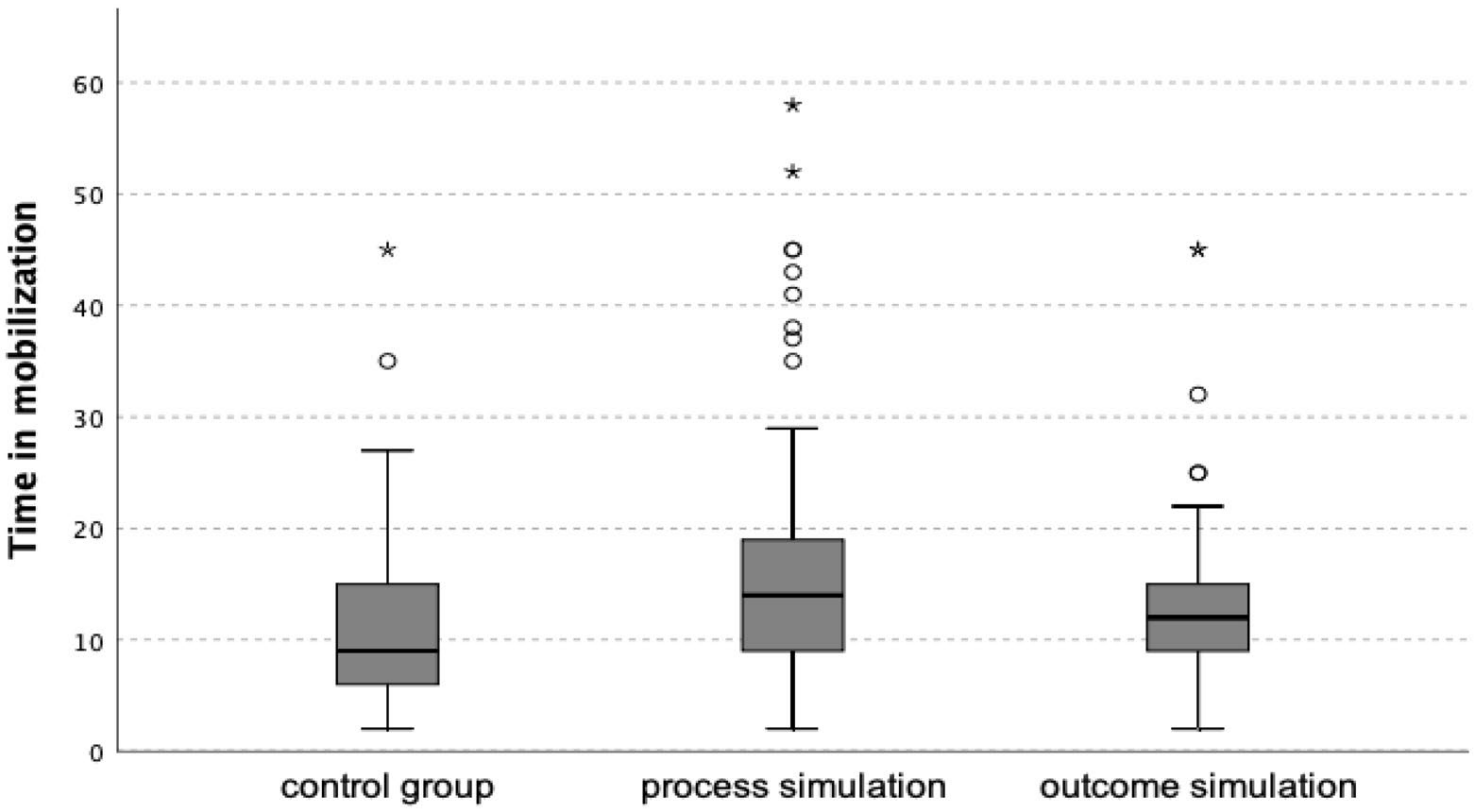
Distribution of time spent in mobilization in the control, process simulation, and result groups.

Because the distribution of the time spent during mobilization was far from normal and the dependent variable had an ordinal character, we used a nonparametric test for our analyses. The omnibus Kruskal–Wallis H test yielded significant differences between the distribution of responses in the three groups, χ^2^(2, *N* = 137) = 9.27, *p* < 0.05. Dunn’s post-hoc analyses with Bonferroni correction showed that participants in the process-simulation group (*Mdn* = 14) spent significantly more minutes in verticalization than participants in the control group (*Mdn* = 9), *U* =  − 3.02, *p* < 0.01. The time spent in mobilization in the result-simulation group (*Mdn* = 12) did not differ from the control group (*Mdn* = 9), nor did it differ from the simulation group (*Mdn* = 14). The distribution of time spent in mobilization in each study group is shown in Fig. 2.

Finally, we tested how anxiety as a state and pain affected the effectiveness of simulations on time spent in verticalization. We performed an ordinal regression in which we entered dummy coded manipulation and anxiety after the simulation, anxiety as a trait or pain as predictors, and time in verticalization as an outcome variable. The analysis revealed that process simulation was a significant predictor of time spent in verticalization, *W* = 3.75, *p* < 0.05 as was anxiety as a state, *W* = 18.05, *p* < 0.001. Pain was a significant predictor, *W* = 5.91, *p* < 0.05, and the outcome-simulation variable was not significant. The outcome-simulation variable was marginally insignificant, *W* = 2.80, *p* = 0.094.

Overall, process simulation was the most effective in motivating patients to stay mobilized longer. Anxiety and pain also played a significant role in the time spent in mobilization. No side effects or unintended effects were reported by study participants.

## Discussion

This study tested whether the use of psychological intervention such as mental stimulation in women after cesarean section can influence the willingness to verticalize, actual verticalization, and the duration of the first mobilization. We found that simulation groups differed from the control group in the number of women who verticalized and in the duration of verticalization. The process-simulation intervention was the most effective, as almost 12% more patients verticalized in this group than in the control group. Almost 5% more of patients verticalized in the outcome simulation than in the active control group, but this difference was not significant. The simulation interventions also affected the time spent in mobilization. Notably, in the process-simulation group, patients spent 5 min more in mobilization than in the control group.

Our results suggest that the process simulation turned the willingness into action, as most patients from this group stood up and obtained the longest mobilization time. In a different study, which was a theoretical inspiration for the present research, students from the process-simulation group spent the most hours studying before an exam^26^. This result can be explained by the fact that in both groups, the patients visualized their movement (in the process group, it was movement related to standing up and walking, and in the outcome, it was efficient movement after verticalization). Research has shown that imagining our own activity involves the same areas in the brain as during behavioral movement^36^. Even one-time mental training of movement imaging improves motor movements^37^. In our study, in the control group, the patients were deprived of motor training in their imagination.

There was no statistically significant difference in the willingness to verticalize between the study groups. This could have resulted from a short time of mental training performed with only one session. Undoubtedly, the duration of mental exercises increases their effectiveness^26,38^. However, it was not possible to examine the patient earlier, e.g., before cesarean section, and offer her several sessions of mental training.

Following the suggestion of Hagger^39^, who argued for not ignoring psychological factors when designing aid interventions in the area related to physical activity, anxiety as a state was controlled in our study. Specific anxiety of verticalization (as a state) increased the statistical significance of the impact of the effectiveness of the process-simulation group on the willingness to verticalize, the number of patients after verticalization, and also the amount of time spent during mobilization.

Probably, the measurements of the willingness to verticalize conducted twice would more accurately show whether the content of the simulation affected the cognitions and behaviors related to mobilization. Research has shown that people’s initial motivation plays a role in the effectiveness of mental simulations, and that mental simulations concerning reaching health goals are mainly important when the goal is difficult to achieve. For easy goals, there is no significant difference between the research groups^40^. In the study measuring the influence of mental images on the promotion of physical activity, respondents with low readiness for physical activity were selected. This was because it is difficult to demonstrate the effectiveness of simulation in already motivated and active people^41^. The studies showed that a carefully prepared implementation plan affects the readiness to achieve the goal in the areas of healthcare^42,43^.

It seems worthwhile to continue research on the influence of mental simulations for patients with the high anxiety of verticalization and a simultaneous low willingness to verticalize. The initial selection of patients with a high level of anxiety and low willingness to verticalize declared before cesarean section could be used when selecting a group that needs additional support.

The conducted study can be treated as pioneer research, the results of which encourage further exploration of the topic on a larger group of selected patients with both a high level of anxiety of verticalization and low willingness to verticalize. The mental contrasting proposed by Oettingen could be used when planning future interventions for patients with a low willingness to verticalize. Her research showed that the juxtaposition of an unsatisfactory present and a positive future activates the readiness to act^44^. An innovation to Taylor’s classic experiments was the addition of elements of free relaxation to mental training. In medical care, postoperative pain and anxiety management protocols recommend the use of cognitive-behavioral methods such as relaxation and visualization to reduce pain and anxiety^45^.

The measurements of anxiety and pain were supposed to only fulfill a control role in the study. Most studies regarding the effect of relaxation on pain and anxiety are carried out in research schemes, which compare the control group without relaxation and the experimental group with relaxation^12,46,47^. In our study, no typical passive control group without any intervention was created. This decision was influenced by ethical considerations. It was decided that all women participating in the study should have the opportunity to enjoy the benefits of relaxation in the postoperative period. The elements of relaxation were a background for the content of the mental simulations, as is the case with saline for dissolved drugs.

Our study was not free of limitations. First, the mental simulation that was offered to the patients was short and only conducted once. To obtain a stronger mental training effect, the literature recommends introducing multiple training sessions over a longer period. The second limitation was the inability to test the willingness to verticalize before subjecting patients to the intervention. The double measurement of this willingness in dependent groups would allow for a more detailed analysis of the influence of mental simulations when compared to measurements conducted in independent groups.

The literature shows that it is often not easy to demonstrate the advantage of one simulation over another^48^. However, the consistency of the results of our study, which agree with the hypotheses, especially after controlling the patients’ anxiety of verticalization, encourages further exploration of the topic concerning the effective impact of the mental simulation of the process on the first verticalization after a cesarean section. These results give hope that mental simulations can work by creating a bridge between thoughts and goal-directed behavior^26^ in the area of rehabilitation after surgery.

## Conclusions

Collectively, our study was the first account in which the effectiveness of mental simulations was tested in a real setting after cesarean surgery. We found that one-time and short-mental simulation training can effectively motivate patients for their first verticalization after cesarean section surgery. Our study also specified the importance of anxiety that patients experienced before their first verticalization. The level of anxiety may affect the effectiveness of interventions; therefore, it is recommended to have it in mind at the postoperative care and preferable to measure with simple questions.

## Supplementary Information

Supplementary Information.

## Data Availability

All data generated or analysed during this study are included in this published article.
