# Supplementary material for: Effectiveness of mental simulations on the early mobilization of patients after cesarean section: a randomized controlled trial
Source: Sci Rep. 2021 Nov 22;11:22634. doi: 10.1038/s41598-021-02036-1 (PMC8608872; doi:10.1038/s41598-021-02036-1)
Supplement: Supplementary file 1 — Supplementary Information. [file 41598_2021_2036_MOESM1_ESM.docx]

Appendix for the manuskrypt: Manuscript: Effectiveness of mental simulations on the early mobilization of patients after cesarean section: a randomized controlled trial.

**PARTICIPANTS**

Most patients 126 (84.0%) had higher education, and the remain, in the decreasing order, had secondary education - 19 (12.7%), primary education - 2 (1.3%), vocational education - 2 (1.3%), and 1 (0.7%) had lower secondary education. Most patients (n=71, 47.3%) underwent the first cesarean section, 64 (42.7%) the second, 14 (9.3%) the third, and 1 (0.7%) the fourth. Most cesarean sections were planned (n=127, 84.7%); 23 (15.3%) were emergency.

The subject of the present work is beyond the description of the impact of an intervention on the level of pain and anxiety verticalization. The presentation of their levels characterizes the study group in the context of the psycho-physical comfort. Before the intervention, the average pain in the study group was 2.25±1.67, after the intervention 2.95±2.87, during verticalization 3.58±1.96. The average anxiety before the intervention was 3.54±2.88 and after intervention, it was 2.95±2.87. Anxiety as a trait was 36.17±6.9.

**PROCEDURE**

The table below presents all variables measured in the described study.

Table 1: Measurements of variables during the study

| Variable | T0 measurement (upon arrival at the recovery room)  N=150 | T1 measurement (before listening to the recording)  N=150 | T2 measurement (after listening to the recording)  N=150 | T3 measurement (after verticalization and return to bed)  N=137 |
| --- | --- | --- | --- | --- |
| Pain | - | X | X | X |
| Trait anxiety | X | - | - | - |
| Anxiety of verticalization | - | X | X | - |
| Willingness of verticalization | - | - | X | - |
| Verticalization | - | - | X | - |
| Duration of mobilization | - | - | - | X |

“X” – presence of the measurement; “-” – lack of measurement in the study.

**CONTENT OF MANIPULATIONS**

**Recording for the control group**
Fragment of the book “Little Prince” by Antoine de Saint-Exupéry [1].

“Close your eyes, take a deep breath, and feel relaxed. While listening to an excerpt from Antoine de Saint-Exupéry’s book ‘The Little Prince,’ see the world through the eyes of the main character.

But one day, from a seed blown from no one knew where, a new flower had come up; and the little prince had watched very closely over this small sprout, which was not like any other small sprouts on his planet. It might, you see, have been a new kind of baobab. The shrub soon stopped growing and began to get ready to produce a flower. The little prince, who was present at the first appearance of a huge bud, felt at once that some sort of miraculous apparition must emerge from it. But the flower was not satisfied to complete the preparations for her beauty in the shelter of her green chamber. She chose her colors with the greatest care. She adjusted her petals one by one. She did not wish to go out into the world all rumpled, like the field poppies. It was only in the full radiance of her beauty that she wished to appear. Oh, yes! She was a coquettish creature! And her mysterious adornment lasted for days and days.

Then one morning, exactly at sunrise, she suddenly showed herself. And, after working with all this painstaking precision, she yawned and said:

- Ah! I am scarcely awake. I beg that you will excuse me. My petals are still all disarranged...

- But the little prince could not restrain his admiration:

- Oh! How beautiful you are!

- Am I not? - The flower responded, sweetly. And I was born at the same moment as the sun...

The little prince could guess easily enough that she was not any too modest—but how moving and exciting—she was!

Please imagine for a few minutes what the Little Prince felt when he saw his rose for the first time. Feel her scent, see her color, take a look around his world for a few minutes, see all the colors on his planet, feel all the scent.

When you hear the sentence: “Take a deep breath and slowly open your eyes,” complete your imagination and slowly open your eyes.”

**Recording for the process simulation group**

“Close your eyes. Step-by-step imagine the process of the verticalization that you will go through shortly: You are lying in bed, which is in a safe place for you. This may be a place that you have been to, or would like to be. It is important that it is a place where you can rest. You are covered with a duvet, and you feel warm. You are surrounded by pleasant, familiar smells (it can be the smell of familiar perfumes, a dying fire, cooking dinner, or freshly brewed coffee). You hear familiar, friendly sounds (for example, the sound of the sea and waves, the rustle of trees and leaves, or maybe the laughter of children, or the sound of your favorite music). Along with the smells and sounds, refreshing energy flows into you, which you breathe in with all of yourself, and thus energize every cell in your body. You are breathing calmly, and your entire body, arms, and stomach area feel relaxed. You feel that with each breath and the energy flowing from the smell and sounds, your body de-stresses and gains strength at the same time. With renewed energy, you decide to move your hands, neck, and feet. You turn slowly to the side, and then gently rise to a sitting position. If at any point, you feel tension or pain, stop for a moment, relax, and breathe. You can also drink some water. As you breathe and relax, you will gain new strength and energy. You feel stronger, you still feel pain, but you sit up in bed. You decide to continue, and you get up, breathe, and relax. You know what path you have to follow, and you know what work you have to do. Please practice the process of verticalization in your imagination a few more times. When you hear the phrase ‘Take a deep breath and slowly open your eyes,’ stop imagining and slowly open your eyes.”

**Recording for the outcome simulation**

“Close your eyes. Imagine that you have regained your full physical fitness after your cesarean section surgery: you are free and proud of yourself, and you enjoy your fitness. You feel happiness and satisfaction. You are in a perfect place for yourself, where you feel safe. This may be a place you have been to before, or would like to be. It is important that it is a place where you can rest. It may be a warm house, or the bosom of nature, such as a forest, meadow, mountains, or sea. You can experience pleasant familiar smells (it can be the smell of familiar perfumes or flowers, a dying out fire, cooking dinner, or freshly brewed coffee). You hear familiar friendly sounds (for example, the sound of the sea and waves, the rustle of trees and leaves, or maybe the laughter of children, or the sound of your favorite music). Along with the smells and the sounds, refreshing energy flows into you. You breathe it in with all of yourself, thus energizing every cell in your body. You breathe calmly, relaxing your entire body, arms, and stomach area. You feel like with each breath and the energy flowing from the smell and sounds, your body relaxes and gains strength at the same time. You are calm and content. You can do whatever you want; you are fit and light. You can take a walk, take a bath, or eat anything you want. Feel the taste and smell of this dish. Now, please think for a few more minutes about yourself as a completely fit and healthy person, and about the place where your imagination took you. Feel the smells and tastes that you recalled. When you hear the phrase ‘Take a deep breath and slowly open your eyes,’ stop imagining and slowly open your eyes.”

**References**

1. de Saint-Exupéry A. Mały Książę. 2008. https://www.odaha.com/antoine-de-saint-exupery/maly-princ/maly-ksiaze. Accessed 25 October 2020.
